# COVID-19, Blood Lipid Changes, and Thrombosis

**DOI:** 10.3390/biomedicines11041181

**Published:** 2023-04-15

**Authors:** Akhlaq A. Farooqui, Tahira Farooqui, Grace Y. Sun, Teng-Nan Lin, Daniel B. L. Teh, Wei-Yi Ong

**Affiliations:** 1 Department of Molecular and Cellular Biochemistry, Ohio State University, Columbus, OH 43210, USA; 2 Department of Biochemistry, University of Missouri, Columbia, MO 65211, USA; 3 Institute of Biomedical Sciences, Academia Sinica, Taipei 11929, Taiwan; 4 Department of Ophthalmology, Yong Loo Lin School of Medicine, National University of Singapore, Singapore 119260, Singapore; 5 Department of Anatomy, Yong Loo Lin School of Medicine, National University of Singapore, Singapore 119260, Singapore; 6 Neurobiology Research Programme, Life Sciences Institute, National University of Singapore, Singapore 119260, Singapore

**Keywords:** COVID-19, secretory phospholipase A_2_, sPLA_2_-IIA, *PLA2G2A*, lysophospholipase D, autotaxin, lysophosphatidic acid, LPA, platelets, neutrophil extracellular traps, NETs, thrombosis, microthrombi, macrothrombi, COVID-19-associated-coagulopathy, CAC, NETs, pneumocytes, brain endothelial cells, stroke, C16:0 ceramide

## Abstract

Although there is increasing evidence that oxidative stress and inflammation induced by COVID-19 may contribute to increased risk and severity of thromboses, the underlying mechanism(s) remain to be understood. The purpose of this review is to highlight the role of blood lipids in association with thrombosis events observed in COVID-19 patients. Among different types of phospholipases A_2_ that target cell membrane phospholipids, there is increasing focus on the inflammatory secretory phospholipase A_2_ IIA (sPLA_2_-IIA), which is associated with the severity of COVID-19. Analysis indicates increased sPLA_2_-IIA levels together with eicosanoids in the sera of COVID patients. sPLA_2_ could metabolise phospholipids in platelets, erythrocytes, and endothelial cells to produce arachidonic acid (ARA) and lysophospholipids. Arachidonic acid in platelets is metabolised to prostaglandin H2 and thromboxane A_2_, known for their pro-coagulation and vasoconstrictive properties. Lysophospholipids, such as lysophosphatidylcholine, could be metabolised by autotaxin (ATX) and further converted to lysophosphatidic acid (LPA). Increased ATX has been found in the serum of patients with COVID-19, and LPA has recently been found to induce NETosis, a clotting mechanism triggered by the release of extracellular fibres from neutrophils and a key feature of the COVID-19 hypercoagulable state. PLA2 could also catalyse the formation of platelet activating factor (PAF) from membrane ether phospholipids. Many of the above lipid mediators are increased in the blood of patients with COVID-19. Together, findings from analyses of blood lipids in COVID-19 patients suggest an important role for metabolites of sPLA_2_-IIA in COVID-19-associated coagulopathy (CAC).

## 1. Introduction

COVID-19 originated in 2019 and has spread around the world [1]. The SARS-CoV-2 virus, which is the cause of COVID-19, shares highly homologous sequences with the severe acute respiratory syndrome coronavirus (SARS-CoV-1), and causes acute and highly lethal pneumonia with clinical symptoms similar to those produced by SARS-CoV-1 or MERS-CoV infection [2]. It is a single-stranded coronavirus with a positive-sense RNA (+ssRNA) genome of approximately 26–32 kb and is currently the largest known genome size for an RNA virus [3]. SARS-CoV-2 is characterised by the presence of spike proteins that project from its surface [4]. The common symptoms of COVID-19 are fever, cough, sore throat, breathlessness, and fatigue. Other symptoms include sputum production, myalgia or arthralgia, chills, vomiting, and nasal congestion. There is a substantial increase of acute phase reactants in the blood, suggesting a dysregulation of the inflammatory host response. The latter may cause an imbalance between pro- and anti-inflammatory mediators, leading to the recruitment and accumulation of leukocytes in tissues including the lungs and producing acute respiratory distress syndrome (ARDS) [5]. SARS-CoV-2 infection of cells occurs through the binding of angiotensin-converting enzyme 2 (ACE2). Virus binding to ACE2 induces conformational changes in the S1 subunit of its spike protein and exposes the S2′ cleavage site in the S2 subunit. The S2′ site is then cleaved by a protease to expose a peptide within the spike protein that is able to attach to and induce fusion of the viral envelope with the host cell membrane, thus facilitating infection of the cell. For a recent review, see [6].

## 2. SARS-CoV-2 and Induction of Cytokine Storm

SARS-CoV-2 infection promotes the overproduction of inflammatory cytokines with a wide range of biological activity. These cytokines drive positive feedback on immune cells and recruit them to the sites of inflammation. This ‘cytokine storm’ is a life-threatening systemic inflammatory syndrome involving elevated levels of circulating cytokines and immune cell hyperactivation. These processes can lead to COVID-19-associated coagulopathy or thrombosis [7]. Activation of Toll-like receptors (TLRs) by SARS-CoV-2 triggers a biochemical cascade beginning with the generation of pro-IL-1 cleaved by caspase-1, followed by inflammasome activation [8,9]. The NOD-like receptor (or nucleotide-binding domain and leucine-rich repeat containing receptor; NLR) family pyrin domain containing 3 (NLRP3) inflammasomes are large multimolecular complexes that control the activation of caspase-1, which in turn regulates the maturation of IL-1β and IL-18. IL-1β is a pro-inflammatory cytokine that induces local and systemic inflammation and a febrile reaction in response to infection [10,11]. Major cytokines that are overexpressed during SARS-CoV-2 infection include tumour necrosis factor-α (TNF-α), interleukins (IL-1β, IL-6), interferon-γ (IFN-γ), colony stimulating factors (CSF), the chemokine family (CXCL10, CXCL8, CXCL9, CCL2, CCL3, and CCL5), growth factors, and others. These mediators can be divided into pro-inflammatory mediators (such as IL-1β, IL-6, IL-12, TNF-α, and IFN-γ) and anti-inflammatory mediators (such as IL-4, IL-10, IL-13, and TGF-β). The exact mechanism of ARDS in COVID-19 patients is not fully understood, although excessive production of pro-inflammatory cytokines is probably a major contributing factor [4,12,13]. Cytokines and chemokines attract macrophages, neutrophils, and T cells in the lungs [14], and microglia in the brain [15]. The cytokine storm in severe COVID-19 could cause widespread dysregulation of the host immune defence, endothelial dysfunction, damage to the vascular barrier, and diffuse alveolar damage, leading to multi-organ failure and ultimately death [16]. Cytokine storms could also result in hypercoagulation of the blood and thromboses [17]. These effects suggest that targeting the cytokine storm with anti-inflammatory drugs during the management of COVID-19 patients may improve survival rates for severe COVID-19 patients and reduce mortality.

## 3. COVID-19-Associated Coagulopathy (CAC)

Patients with COVID-19 have a higher frequency and severity of clotting events as compared with diseases caused by other common respiratory viral infections. These can manifest as microthrombi and macrothrombi and result in damage to multiple organs, such as the lungs, heart, kidney, and brain (reviewed in [18]). Coagulopathy is associated in most cases with elevated plasma levels of D-dimer, C-reactive protein, P-selectin, and fibrinogen [19,20]. D-dimer is generated by plasmin cleavage of cross-linked fibrin and is therefore a marker of both coagulation events and fibrinolysis. Elevated D-dimer levels in patients with COVID-19 are accompanied by only occasional prolongation of the prothrombin and activated partial thromboplastin times [4]. These haematological changes are not consistent with classical disseminated intravascular coagulation [21], but rather suggest a different aetiology for CAC that could include disturbances in fibrinolysis [22]. Abnormal coagulation parameters are often associated with poor prognosis in patients [23]. Variants of COVID-19 differ in their effects on clotting. Although clotting parameters associated with the omicron variant of SARS-CoV-2 are significantly raised over those of healthy matched controls, their levels are significantly lower than those seen with more severe variants such as beta and delta [24]. Since the omicron variant appears to be far more transmissible but less virulent than the beta or delta variants, the less extensive clot formation appears to correlate with the reduced virulence of this variant [24]. These results support the notion that the ability to induce micro- and macrothrombi plays an important role in the virulence of COVID-19.

Hypofibrinolytic state and high thrombin generation play major roles in SARS-CoV-2-associated thrombosis [25]. One of the prominent features of COVID-19-associated coagulation is changes in the plasma levels of plasminogen, plasmin, and D-dimer. Plasminogen is the precursor of plasmin, which lyses fibrin clots to form fibrin degradation products and D-dimer. The conversion to active protease is mediated by tissue-type (tPA) and urokinase-type (uPA) plasminogen activators. Several studies have reported increases in plasminogen activator inhibitor (PAI-1) in patients with COVID-19. This could lead to reduced plasminogen activation, decreased formation of plasmin, and a lower rate of dissolution of blood clots [26,27]. PAI-1 and its cofactor, vitronectin, are significantly elevated in patients with COVID-19 as compared with those with a non-COVID-19 respiratory infection or healthy control groups. Moreover, PAI-1 and tissue plasminogen activator (tPA) are found in patients with more severe COVID-19 disease [26]. It is also reported that the fibrin produced in COVID-19-associated coagulopathy has features of amyloid [28], which is more resistant to fibrinolysis, and associated with anti-plasmin [27,28]. Mass spectrometry showed that when spike protein S1 is added to healthy platelet-poor plasma, it results in structural changes to fibrin(ogen), complement 3, and prothrombin, and these become substantially more resistant to trypsinization [29].

Factors within the bloodstream, as well as those in the vessel walls or around the vessel walls, likely play important roles in COVID-19 associated coagulopathy. Contributing factors within the bloodstream could include the formation of neutrophil extracellular traps (NETs). In response to injury, neutrophils generate threads of chromatin covered with granule-derived peptides and proteolytic enzymes, or NETs [30]. The latter has a large net-like structure in which pathogens may be trapped [31]. NETs, however, are also involved in pathophysiological mechanisms ranging from inflammation to thrombosis [32]. Neutrophils were found in autopsy specimens from the lungs of a patient who succumbed to COVID-19. Extensive neutrophil infiltration was found in pulmonary capillaries, with acute capillarities with fibrin deposition and extravasation into the alveolar space [33]. The sera of patients with COVID-19 have elevated levels of cell-free DNA, myeloperoxidase-DNA (MPO-DNA), and citrullinated histone H3 (Cit-H3); the latter two are specific markers of NETs [34]. MPO-DNA is associated with both cell-free DNA and the absolute neutrophil count, while Cit-H3 is correlated with platelet levels [34]. Myeloperoxidase (MPO), produced by neutrophils, catalyses the formation of reactive oxygen intermediates, including hypochlorous acid (HOCl). The latter plays an important role in microbial killing but could also be a mediator of tissue damage [35], especially in the tunica intima of blood vessels adjacent to the bloodstream. Another NET by-product implicated in COVID-19 pathogenesis is elastase. The latter can accelerate virus entry and induce hypertension, thrombosis, and vasculitis [36], and might also damage elastic lamellae in the tunica media of blood vessels, as well as elastic fibres in the interalveolar septa of the lungs. This could lead to reduced elastic recoil of blood vessels and lungs. In addition to neutrophils, platelets have been shown to induce NET formation, and, in turn, NET’s components regulate neutrophil and platelet function [37]. Blood lipids that participate in platelet activation include platelet activating factor (PAF). The latter is an ether phospholipid and a potent chemical mediator of inflammation [38]. PAF is produced by cells involved in host defense, and its biological actions bear similarities with COVID-19 disease manifestations [39]. Increased levels of PAF have been found in the blood of moderate COVID-19 patients [40]. In addition to PAF, lysophosphatidic acid has been found to promote thrombus stability by inducing the rapid formation of NETs [41].

Factors within the vessel walls, or around the vessels could also be a source of increased coagulation in COVID-19. Dysfunction of the vascular endothelium is thought to be a major contributor to the pathogenesis of COVID-19 vasculopathy [42,43,44]. Injury to endothelial cells could lead to increased expression of pro-coagulation factors including von Willebrand factor, thromboxane A2, thromoplastin, Factor V, PAF, and plasminogen activator inhibitor [45]. In addition, loss or damage to endothelial cells might result in exposure of tissue factor (TF), leading to activation of plasma factor VII/VIIa (FVII/FVIIa) [45]. Injury to cells around the blood vessels might also affect endothelial cells and result in coagulopathy. Closely adherent to endothelial cells in the lungs and separated only by a basement membrane are lung epithelial cells, including flattened Type I pneumocytes and Type II pneumocytes, which secrete surfactant and normally express ACE2 [46,47]. After SARS-CoV-2 infection, there is induction of ACE2 expression in the lungs [46]. Induction of ACE2 enzyme is normally part of an anti-inflammatory response through the ACE2/Angiotensin-(1-7)/Mas receptor signalling axis [48], but in the case of COVID-19, could facilitate further SARS-CoV-2 infection of pneumocytes [46]. The SARS-CoV-2 spike protein S1 subunit induces pro-inflammatory responses via Toll-like receptor 4 signalling in murine and human macrophages [49] and could induce inflammation and injury to lung epithelial cells. In turn, this could lead to damage to the adjacent endothelial cells, loss of endothelial barrier function, oedema, and reduced gaseous exchange across the alveolar-capillary membrane (blood–gas barrier). Endothelial injury could also lead to increased microthrombi formation in capillary beds around the alveoli [50]. This could exacerbate the difficulty in gaseous exchange and compromise the supply of nutrients to pneumocytes and endothelial cells.

Increased immune reaction in and around the blood vessel walls could lead to upregulation of lipolytic enzymes, including intracellular cytosolic phospholipase A_2_ (cPLA_2_) and secretory phospholipase A_2_ (sPLA_2_) [51,52]. Secreted sPLA_2_ could leak into the bloodstream from the affected region, and affect other vascular beds and tissues in more distant locations.

## 4. COVID-19 and Changes in Phospholipases A_2_

Proinflammatory cytokines, such as IL-1β and TNF-α may induce the de novo synthesis of cPLA_2_ [53], which catalyses the breakdown of membrane phospholipids to produce a free fatty acid (arachidonic acid, ARA), and a lysophospholipid. In turn, ARA can be metabolised by cyclooxygenases (COX) to produce inflammatory lipid mediators such as prostaglandins [54]. In addition to the calcium-dependent cPLA_2_, other isoforms of PLA_2_ such as the calcium-independent iPLA_2_ and secretory sPLA_2_ (sPLA_2_) are involved in inflammatory events [55]. These PLA_2_s have different molecular structures and cellular localizations and produce lipid mediators with diverse functions. In a study on an animal model in which ischemic stroke was induced by occlusion of the middle cerebral artery, an increase in sPLA_2_-IIA mRNA was found in the peri-infarct area [56]. Other studies have well-demonstrated the involvement of both cPLA_2_ and sPLA_2_ in models of stroke [54].

Increased sPLA_2_-IIA was recently shown to parallel several indices of disease severity in patients with COVID-19. A decision tree generated by machine learning has identified sPLA_2_-IIA level as a *central node* in the stratification of patients who died from COVID-19 [57]. Increases in D-dimer, a protein fragment produced during dissolution of blood clots, together with the pro-inflammatory marker, C-reactive protein (CRP), ferritin, TNF-α, IL-1β, IL-6, and IL-13, as well as sPLA_2_-IIA activity, were also found in patients with COVID-19 compared to normal persons [58]. These results indicate a link between cytokine storm and sPLA_2_-IIA activity in patients with COVID-19 [58].

sPLA_2_ level is also correlated with the severity of COVID-19 and acute multisystem inflammatory syndrome (MIS-C) in children [59]. In addition, comprehensive analyses of the plasma proteome of more than 1400 proteins in children with COVID-19 showed significant overlap in protein signatures between severe COVID-19 and MIS-C, as well as the inflammatory syndromes, macrophage activation syndrome, and thrombotic microangiopathy [60]. Interestingly, sPLA_2_-IIA was found to be *an important marker of MIS-C* that associates with thrombotic microangiopathy [60]. These findings indicate a close relationship between plasma sPLA_2_-IIA levels and thromboses in patients with severe COVID-19 or MIS-C.

sPLA_2_-IIA in human atherosclerotic lesions has been implicated in the initiation, progression, and maturation of atherosclerosis, which is a risk factor for thrombosis, including stroke [61]. Patients with metabolic syndrome showed strikingly higher levels of endothelial activation molecules that were correlated with increased serum sPLA_2_-IIA protein levels and activity [62]. Lung microvascular endothelial cells are highly sensitive targets for the direct action of extracellular sPLA_2_, and a specific pERK inhibitor, U0126, was found to prevent sPLA_2_-induced chemokine upregulation [63]. Another study showed the ability of a sPLA_2_ inhibitor, indoxam, to suppress low-density lipoprotein (LDL) modification and associated inflammatory responses in TNFα-stimulated human endothelial cells [64].

Substrates for serum sPLA_2_ could include phospholipids that are derived from the cellular membranes of platelets, erythrocytes, endothelial cells, and possibly gut bacteria that have entered the bloodstream. Platelets are capable of releasing mitochondria into the bloodstream either as vesicle-enclosed microparticles or as free organelles [65]. The released mitochondrial membranes could serve as substrates for circulating sPLA_2_-IIA. Another possible source of substrate for circulating sPLA_2_-IIA could be the plasma membrane of erythrocytes. Although normal erythrocytes are apparently not affected by sPLA_2_-IIA, at high levels, such as those observed under inflammatory conditions, phosphatidylserine-exposing erythrocytes could undergo haemolysis and generate LPA [66].

In addition to sPLA_2_-IIA, sPLA_2_-IID has been found to contribute to age-related susceptibility to SARS-CoV-1 infection in mice [67] and plays a key role in coronavirus-specific antibody production in middle-aged mice [68]. sPLA_2_-IID has also been shown to be necessary for the virulence of SARS-CoV-2 [69]. COVID-19 disease severity was significantly reduced in aged mice that lacked sPLA_2_-IID or the prostaglandin D2 receptor DP1; treatment with a DP1 antagonist, asapiprant, protected these mice from lethal COVID-19 infection [69].

An increase in another sPLA_2_ isoform encoded by the *PLA2G7* gene, lipoprotein-associated phospholipase A_2_ (Lp-PLA_2_), has also been detected in the sera of patients with COVID-19 [70]. This enzyme is associated with low-density lipoprotein and is involved in the pathogenesis of cardiovascular disease [70].

A compound, 2-oxoamide GK241, has recently been developed that is a dual inhibitor of sPLA_2_-IIA and the SARS-CoV-2 main protease, and could be a promising candidate for combating COVID-19 [71].

**Figure 1:**
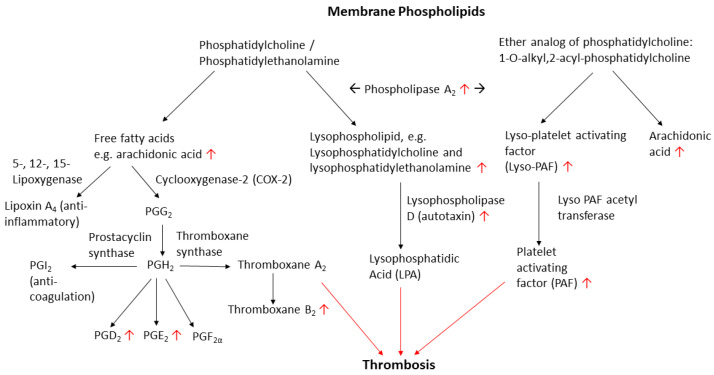
Phospholipid metabolites and pathways leading to thrombosis. Red arrows indicate enzymes and metabolites that are increased in the blood of COVID-19 patients (for details, please see text).

Phospholipid metabolites that are produced due to PLA_2_ activity and increased in COVID-19 are summarised in Figure 1.

## 5. COVID-19-Associated Changes in Arachidonic Acid (ARA) and Docosahexaenoic Acid (DHA)

Increased levels of sPLA_2_ could contribute to greater levels of arachidonic acid (ARA) in the serum of patients with COVID-19. Dysregulation of lipid metabolism together with pathological inflammation has been detected in patients with COVID-19 [72]. In addition, alterations of lipids and metabolites were correlated with the course of disease in COVID-19 patients [73]. Large-scale plasma analyses of patients with COVID-19 showed changes in phosphatidylcholine and phosphatidylethanolamine, as well as ARA and oleic acid, which correlate with disease severity [74]. Semi-targeted lipidomic analyses of 126 COVID-19-positive patients identified ARA, lysophosphatidylethanolamine, acylcarnitine, and oxylipins as the most altered lipid species in COVID-19 patients compared to healthy volunteers [75]. Likewise, targeted metabolomic analyses showed ARA, sphingolipid, tryptophan, tyrosine, glutamine, and arginine metabolism to be the most affected pathways in hospitalised patients with COVID-19 [76]. SARS-CoV-2 infection increased plasma and tracheal aspirate levels of ARA, 5-hydroxy-6E,8Z,11Z,14Z-eicosatetraenoic acid,11-hydroxy-5Z,8Z,12E,14Z-eicosatetraenoic acid, and acetylcholine [77]. High plasma levels of non-esterified polyunsaturated fatty acids have been regarded as a specific feature of patients with severe COVID-19 pneumonia. Among hospitalised patients with severe pneumonia, COVID-19 is associated with higher concentrations of non-esterified fatty acids, especially ARA and linoleic acid [78]. One study, however, reported lower levels of ARA and linoleic acid in children with COVID-19 and associated MIS-C, which might be attributed to increased metabolism of these fatty acids [79]. Lower levels of ARA but higher levels of oxylipins derived from non-enzymatic peroxidation of polyunsaturated fatty acids have also been reported in the plasma of COVID-19 patients in an intensive care unit [80]. Taken together, the above findings indicate perturbations of ARA and/or its metabolites in severe COVID-19 cases (Figure 1).

Arachidonic acid (ARA) and docosahexaenoic acid (DHA) are important polyunsaturated fatty acids released from phospholipases A_2_ [81]. A Yin-Yang mechanism regulates the metabolism of these two fatty acids in the central nervous system, resulting in inflammatory vs. protective responses [82]. In contrast to ARA, which is mostly associated with pro-inflammatory mediators, the omega-3 fatty acid DHA is metabolised to resolvins, protectins, and maresins, which have anti-inflammatory and pro-resolving properties [83]. Observations support the notion that DHA may alleviate the severity of symptoms of COVID-19 [84].

Higher amounts of pro-inflammatory and pro-thrombotic lipid mediators are present in the plasma of COVID-19 patients, as compared with healthy subjects [85]. Conversely, reduced concentrations of specialised pro-resolving mediators have been detected in the sera of severe COVID-19 patients [86]. These results suggest an imbalance that favours the pro-inflammatory over the anti-inflammatory pathways in COVID-19. Supplementation of COVID-19 patients with moderate dosages of omega-3 fatty acids has resulted in improvement of inflammation-related clinical symptoms in a randomised clinical trial [87].

## 6. COVID-19-Associated Changes in Serum Glycerophospholipids and Proinflammatory Lipid Mediators

### 6.1. Changes in Glycerophospholipids and Sphingolipids/Ceramide

Changes in glycerophospholipids have been a consistent finding in the serum lipids of patients with COVID-19. A recent study showed significant increases in the levels of serum phospholipids, including sphingomyelins and phosphatidylcholines, in the serum of COVID-19-positive patients as compared with COVID-19-recovered individuals [88]. Likewise, fatty acid and glycerophospholipid levels were higher in severe COVID-19 patients than in recovered patients [89]. Analyses of plasma from COVID-19 patients showed changes in 54 lipids belonging to 12 lipid classes. Of these, glycerophospholipids, sphingolipids, and ether lipids were the most significantly perturbed [90]. Plasma metabolite profiles of COVID-19 survivors with abnormal pulmonary function were different from healthy subjects with normal pulmonary function. These alterations were associated with disease severity and involved mainly glycerophospholipid and amino acid metabolic pathways [91]. Consistently, metabolomic pathway analyses to identify potential molecular signatures to discriminate between severe and non-severe COVID-19 revealed that COVID-19 significantly affected glycerophospholipids and metabolic pathways involving linoleic acid [92]. Abnormally high levels of ketone bodies (acetoacetic acid, 3-hydroxybutyric acid, and acetone) and 2-hydroxybutyric acid, a marker of oxidative stress, have also been detected in the sera of hospitalised patients with COVID-19 [93]. COVID-19 patients in the intensive care unit showed elevated levels of oxylipins derived from polyunsaturated fatty acids by non-enzymatic peroxidation or soluble epoxide hydrolase [80].

A study involving 215 serum samples from COVID-19 subjects showed increased lysophosphatidylinositol and C16:0 ceramide levels but decreased phosphatidylinositol, C18:1 ceramide, dihydrosphingosine, lysophosphatidylglycerol, and phosphatidylglycerol levels, compared to patients with infectious diseases other than COVID-19 [94]. The increase in C16:0 but decrease in C18:1 ceramide is interesting, as it implies increased expression of the serine palmitoyltransferase long chain 3 subunit (SPTLC3) [95,96], but decreased expression of the SPTLC2 subunit, of the ceramide biosynthetic enzyme serine palmitoyltransferase (SPT). An imbalance between the levels of C16 and C18 ceramides could lead to changes in membrane fluidity [97] and function.

### 6.2. Thrombxane A_2_ (TxA_2_)

ARA that is released by the action of PLA_2_ may be metabolized by cyclooxygenases (COXs) to PGG_2_ and PGH_2_, and subsequently by thromboxane synthase to produce TxA_2_. The latter has blood coagulative and vasoconstrictive properties [98]. In COVID-19 pneumonia, there is a massive increase in ARA and associated lipid mediators resulting from cyclooxygenase metabolites—notably TxB_2_ ≫ PGE_2_ > PGD_2_ in the lungs and 11-dehydro-TxB_2_ in the systemic circulation [99]. Increased thromboxane levels in the systemic circulation could lead to the formation of platelet-neutrophil aggregates and a faster rate of platelet aggregation in COVID-19 patients [99]. TxA_2_ that is released from platelets is capable of stimulating platelet activation and aggregation. Moreover, TxA_2_ is a known vasoconstrictor [98], and can stimulate the thromboxane prostanoid receptor (TP) that is induced by the cytokine IL-1 [100].

**Figure 2:**
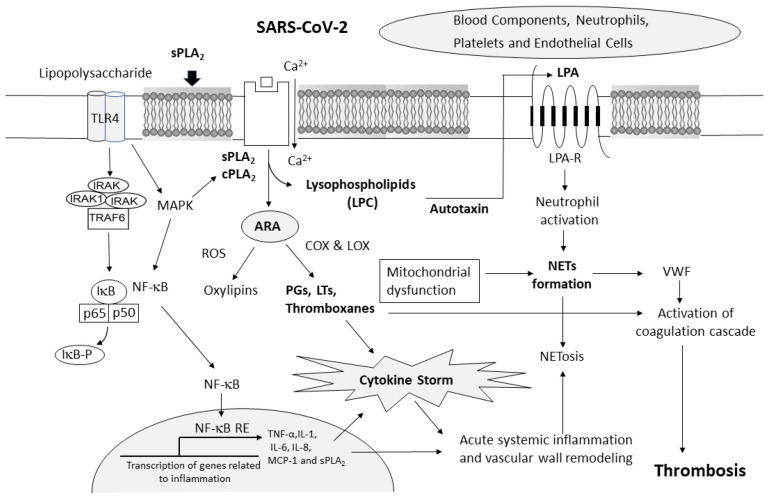
Concept of lysophosphatidic acid generation, the release of NETS, and how these processes contribute to inflammation and increased thrombosis risk following SARS-CoV-2 infection.

Preliminary evidence indicates that a dual blocker of TP and the PGD2 receptor, ramatroban, can produce rapid relief from dyspnoea and hypoxemia in patients with COVID-19 [101,102]. In another study, treatment of four COVID-19 outpatients with ramatroban resulted in rapid relief of dyspnoea and hypoxaemia within 12–36 h and complete resolution over 5 days [103] (Figure 1 and Figure 2).

### 6.3. Leukotriene A4 (LTA4) and Lipoxin A4

LTA4 is an enzymatic product of 5-lipoxygenase (ALOX5), expressed during inflammation in smooth muscle cells, platelets, and the vascular endothelium [104]. Moderate and severe COVID-19 patients were reported to have altered abundances of immune regulatory and proinflammatory lipid mediators, including increased levels of products of ALOX5 and cytochrome p450 but decreased levels of products of ALOX12 and COX-2 [105]. Montelukast, a leukotriene receptor antagonist, has recently been found to reduce platelet activation in plasma from COVID-19 patients. This compound could have potential as an auxiliary treatment for the COVID-19-associated hyperinflammatory/thrombotic state [106].

The actions of the different lipoxygenase enzymes on the production of pro-inflammatory vs. anti-inflammatory lipid mediators are complex. Arachidonic acid can also be metabolised by 5-, 12-, and 15-lipoxygenases to lipoxin A4, which has anti-inflammatory and anti-oxidative stress properties [107].

### 6.4. Prostaglandin E2 (PGE_2_)

PGE_2_ levels in the blood were markedly elevated and correlated positively with disease severity in patients with COVID-19 [108]. SARS-CoV-2 could induce PGE_2_ and secretion in infected lung epithelial cells by upregulating COX-2 and downregulating the prostaglandin-degrading enzyme 15-hydroxyprostaglandin-dehydrogenase [108]. Increased levels of PGE2, galectin-1, and galactin-3 have been detected in patients with COVID-19 compared with healthy controls [109].

### 6.5. Prostacyclin (PGI_2_)

In addition to pro-inflammatory mediators, ARA can be metabolised to PGH_2_ and thereafter to PGI_2_, which has anti-coagulation and vasorelaxant properties. Iloprost, an analogue of PGI_2_, was used for the treatment of foot ischemia after surgical thromboembolectomy in a COVID-19 patient. Unexpectedly, this also resulted in an improvement in respiratory symptoms, and high-resolution computed tomography of the lung showed significant regression of diffuse pulmonary ground-glass opacity. Results suggest that iloprost could have potential for treating COVID-19 [110].

A pilot study of 80 severe COVID-19 patients who received a 72-h infusion of 1 ng/kg/min PGI_2_ showed a trend towards a beneficial effect, although outcomes such as “days alive without mechanical ventilation” were not significantly different from placebo [111]. Prostacyclin infusion to COVID-19 patients resulted in decreased endothelial glycocalyx shedding (syndecan-1) at 24 h compared to placebo-treated controls, suggesting a protective effect on endothelial cells [112].

## 7. COVID-19-Associated Changes in Lysophospholipids

The other product of PLA_2_ action on phospholipids apart from ARA is lysophospholids. Analyses of the plasma of COVID-19 patients using metabolomic and proteomic approaches showed that 30 out of 33 metabolites analysed differed significantly between patients and healthy controls [113]. LysoPC and LysoPE levels were significantly higher in COVID-19 patients at high risk for thromboses than in patients at low risk (D-dimer ≤ 900 U/mL) [113].

These above findings provide additional support for a close relationship between PLA_2_ activity and thromboses in patients with COVID-19.

Another class of lysophospholipids is PAF, which is synthesised in a two-step “re-modelling pathway” during acute or chronic inflammation. The action of PLA_2_ on an ether analogue of phosphatidylcholine, 1-O-alkyl,2-acyl-phosphatidylcholine, results in the release of ARA and 1-O-alkyl-sn-glycerol-3-phosphocholine (lyso-PAF). The latter is then acetylated through the action of acetyl-CoA:lyso-PAF acetyltransferases to produce PAF. Both lyso-PAF and PAF species were found in the plasma of COVID-19 patients [40]. Levels of PAF are higher in mild/moderate COVID-19 patients compared to healthy controls, but levels decrease in individuals with severe/critical disease [40] (Figure 1 and Figure 2). The above findings provide additional support for a close relationship between PLA2 activity and thromboses in patients with COVID-19.

## 8. COVID-19-Associated Changes in Autotaxin

Lysophospholipids produced by the action of PLA_2_ could be metabolised by lysophospholipase D/autotaxin (ATX) and converted to LPA [114,115]. The latter has recently been found to induce the formation of neutrophil extracellular traps (NETS), which promote thrombus formation [41]. Serum ATX levels were correlated with levels of IL-6 and endothelial damage biomarkers, suggesting a relation between the ATX/LPA axis and hyperinflammation and associated vascular dysfunction in patients with COVID-19 [116]. Increased ATX levels in the plasma are also correlated with markers of vascular dysfunction and increased mortality in patients with severe sepsis [117]. Dexamethasone treatment of mechanically ventilated patients result in reduced ATX level, and this might be one of the mechanisms for the therapeutic benefit of the corticosteroid in patients with severe COVID-19 [116].

In addition to serum ATX, endothelial ATX could play a role in local LPA production and atherosclerotic plaque formation in apoE knockout, hypercholesterolaemic mice [118]. Inhibition of ATX by pipedimic acid was shown to decrease bovine endothelial monolayer permeability after anoxia-reoxygenation treatment and reduce the permeability of perfused rat mesenteric post-capillary venules after ischemia reperfusion injury [119] (Figure 1 and Figure 2).

## 9. Lysophosphatidic Acid (LPA) and Platelets in NETs—Contribution to Thrombosis

Evidence supports immunothrombosis, including coagulopathy, thrombopathy, and endotheliopathy, as a mechanism for COVID-19-associated coagulopathy [120]. A hypercoagulable state could result from endothelial damage, complement activation, platelet hyperactivity, release of NETs, activation of the coagulation system, and a hypofibrinolytic state [121,122]. Platelet activation is a major driver of inflammation/thrombogenesis, and von Willebrand factor (vWF) and Platelet Factor 4 (PF4) are deeply involved in the pathogenesis of COVID-19-associated coagulopathy [123,124]. Consequently, platelet hyper-reactivity has been linked to a worse clinical outcome in patients with COVID-19 [125]. It is suggested that PLA_1_ in activated platelets could generate a pool of *sn*-2 lysophospholipids, which undergo acyl migration to yield *sn*-1 lysophospholipids. The latter could then be cleaved by ATX to generate sn-1 LPA species containing 18:2 and 20:4 fatty acids [126]. In addition to platelets, erythrocytes could be a source of LPA. This might involve the action of sPLA_2_ on phosphatidic acid (PA) that is exposed on the outer leaflet of the cell membrane via the action of scramblase [127]. LPA produced by activated platelets could interact with the scramblase TMEM16F in erythrocytes, thus mediating a pro-thrombotic effect [128].

A recent finding that could be particularly relevant to our understanding of COVID-19-associated thrombotic events is the ability for LPA to promote thrombus stability by inducing the rapid formation of NETs [41] (Figure 1). LPA-induced NETs formation could provide a scaffold for plasma protein binding and generate a tissue plasminogen activator (tPA)-resistant blood clot. In turn, LPA-induced NETs could activate platelets to further release LPA [41]. The above could produce a vicious cycle and amplify an immunothrombogenic environment, which is characterised by platelet/NET interactions [129].

Patients with acute pulmonary embolism showed elevated levels of neutrophils, NETs (dsDNA, MPO-DNA, citrullinated histone H3, and nucleosomes), LPA18:1 and LPA20:4, and ATX [124]. ATX and LPA have been found to contribute to increased blood-brain barrier disruption and tissue damage in mouse models of ischemic stroke [130] and might also play a role in COVID-19-related vessel damage.

## 10. COVID-19 and Inflammation in Blood Vessel Walls

In addition to the production of lipid mediators that facilitate the clotting process, it is possible that circulating sPLA_2_-IIA might induce inflammatory changes in blood vessels, and the resultant vasculitis could contribute to thrombus formation. A unique neurologic complication of COVID-19 has been reported in a patient who had extensive cerebral small-vessel ischemic lesions resembling cerebral vasculitis with a combined imaging pattern of ischemia, haemorrhage, and punctuate post-contrast enhancement [131]. Another study showed that among 69 COVID-19 patients, 11 (16%) presented with arterial vessel wall thickening with homogeneous and concentric enhancement, compatible with cerebral vasculitis [132]. Perivascular and intraluminal lymphohistiocytic inflammatory infiltrates consistent with vasculitis have also been reported in post-mortem brain tissue of a previously healthy child with COVID-19 [133].

## 11. Nonenzymatic Effects of sPLA_2_s on the Coagulation Pathway

Other than their functions as enzymes, sPLA_2_s also act as ligands for a wide variety of structurally diverse sPLA_2_ binding proteins. The result of binding could be an increase or a decrease in phospholipolytic activity or effects that are independent of sPLA_2_ enzymatic activity (for a recent review, see [134]). For example, snake venom group IIA-secreted phospholipase A_2_ (SVPLA_2_) has been shown to inhibit blood coagulation through direct binding to human blood coagulation factor Xa (FXa) via a non-catalytic phospholipid-independent mechanism [135]. Little is known about the effects of binding to other types of proteins on blood coagulation. It would be interesting to determine if sPLA_2_-IID (see Section 4 above), which in addition to being induced in inflammatory tissues and capable of producing ARA similar to sPLA_2_-IIA is also a heparin-binding protein [136,137], could have an effect on blood coagulation.

## 12. COVID-19 and Risk of Thrombosis, e.g., Stroke

**Figure 3:**
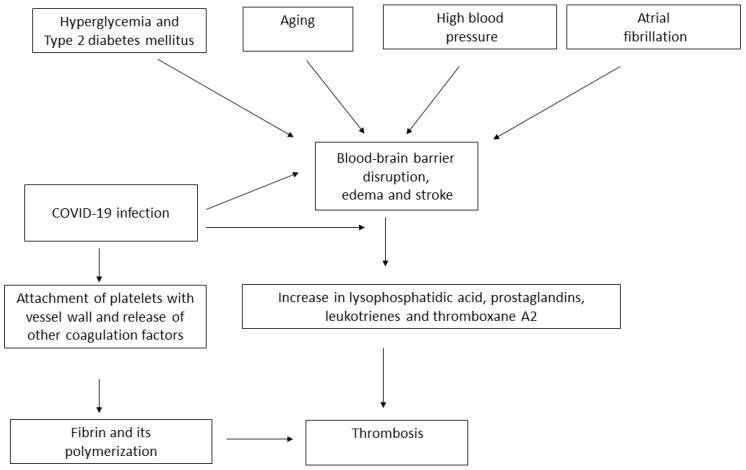
Risk factors for stroke.

Modifiable risk factors for stroke include hypertension, diabetes, heart disease, hypercholesterolemia (atherosclerosis), atrial fibrillation, high alcohol consumption, cigarette smoke, and use of oral contraceptives, whereas non-modifiable risk factors are age, family history of cerebrovascular diseases, gender, and race [138]. Recent studies have indicated that SARS-CoV-2 infection may increase the risk of both ischemic and haemorrhagic stroke [139,140,141] (Figure 3).

SARS-CoV-2 infection may produce a hypercoagulation state and contribute to thrombotic complications, including stroke [139,142,143]. At the molecular level, SARS-CoV-2 infection promotes thrombosis and stroke via modulating the renin-angiotensin system and increasing phospholipid and sphingolipid metabolism through activation of PLA_2_ and sphingolipid degrading enzymes [57,141,144]. To evaluate whether COVID-19 contributes to a higher risk of ischemic or haemorrhagic stroke than just viral respiratory infections, studies have compared the risk of acute ischemic stroke in patients with COVID-19 versus patients with influenza, which is a known risk factor for ischemic stroke [145]. Patients with COVID-19 were found to have a greater risk of ischemic stroke than patients with influenza [146]. Analyses of data from multicentre studies and published cohorts indicate an association between the severity of COVID-19 and an increased risk of acute stroke [147]. Likewise, meta-analysis shows an association between severe COVID-19 and an increased risk of acute ischemic stroke [148]. Patients admitted to the intensive care unit for severe COVID-19 had an increased risk of venous thromboembolic events [149,150]. The frequency of detected stroke in hospitalised COVID-19 patients was 1.1% and was associated with older age and the presence of other stroke risk factors [151]. The most common form of COVID-19-associated stroke was acute ischemic stroke (87.4%), followed by intracerebral haemorrhage (11.6%) [152]. Patients with COVID-19 who developed an acute stroke were older and more likely to have hypertension, diabetes mellitus, coronary artery disease, or severe infection [152]. In addition, many of these patients had elevated levels of D-dimer [153,154]. Antiphospholipid antibodies were also detected in a significant number of cases [154]. The latter could trigger a hypercoagulable state such as that of antiphospholipid syndrome (APS) [155]. Deranged clinical parameters, including altered coagulation profiles, liver function tests, and full blood counts, have been detected in COVID-19 patients who developed stroke as a complication [156].

## 13. Conclusions

The above account suggests a relation between the severity of COVID-19, the extent of phospholipid changes, and thrombosis risks such as stroke. A major focus appears to be the increase in sPLA_2_-IIA and its effect on releasing ARA and lysophospholipids. ARA can interact with COX-2 and LOX to produce lipid mediators such as PGs and TxA_2_ and lead to a cytokine storm. Stimulation of the LPA receptor could lead to activation of neutrophils and subsequently NET formation, which leads to blood coagulation. The above effects involve inflammation and lipid mediators and point towards increased thrombosis risk. A limitation of some of the above studies is that association does not mean causation, and it is unclear whether blood lipid changes are a cause or a consequence of COVID-19 pathophysiology. Further work is necessary to determine whether efforts to reduce inflammation, inhibition of sPLA_2_-IIA, and/or control of lipid mediators could reduce coagulopathy, vasculitis, and the risk of thrombotic events in patients with COVID-19.

## Data Availability

Not applicable.
